# First Real-World Evidence on the Safety and Effectiveness of Lutathera^TM^ for Treating Gastroenteropancreatic Neuroendocrine Tumors (GEP-NETs): Insights from Post-Marketing Surveillance

**DOI:** 10.3390/cancers17182992

**Published:** 2025-09-13

**Authors:** Yong-il Kim, Hoon Young Suh, Yehrim Kang, Hojin Cho, Seung Hyup Hyun, Yoo Sung Song, Seunggyun Ha, Keon Wook Kang

**Affiliations:** 1 Department of Nuclear Medicine, Asan Medical Center, University of Ulsan College of Medicine, Seoul 05505, Republic of Korea; kyi821209@amc.seoul.kr; 2 Theranostics Center, Asan Cancer Institute, Asan Medical Center, Seoul 05505, Republic of Korea; 3 Medical Department, Novartis Korea Ltd., Seoul 07326, Republic of Korea; 4 Department of Nuclear Medicine, Severance Hospital, Yonsei University College of Medicine, Seoul 03722, Republic of Korea; 5 Department of Nuclear Medicine, Samsung Medical Center, Sungkyunkwan University School of Medicine, Seoul 06351, Republic of Korea; 6 Department of Nuclear Medicine, Seoul National University Bundang Hospital, Seoul National University College of Medicine, Seongnam 13620, Republic of Korea; 7 Division of Nuclear Medicine, Department of Radiology, Seoul St. Mary’s Hospital, College of Medicine, The Catholic University of Korea, Seoul 06591, Republic of Korea; seunggyun.ha@catholic.ac.kr; 8 Department of Nuclear Medicine, Seoul National University Hospital, Seoul 03080, Republic of Korea; 9 Cancer Research Institute, Seoul National University College of Medicine, Seoul 03080, Republic of Korea; 10 Bio-MAX Institute, Seoul National University, Seoul 08826, Republic of Korea

**Keywords:** Lutathera, radiopharmaceutical therapy, GEP-NETs, real-world evidence, safety

## Abstract

**Background/Objectives:** This post-marketing surveillance study was conducted to evaluate the safety and effectiveness of [^177^Lu]Lu-DOTA-TATE in patients with GEP-NETs in real-world practice in South Korea. **Methods:** From July 2020 to July 2024, a total of 89 patients from 6 institutions diagnosed with GEP-NETs, as outlined in the approved indication for [^177^Lu]Lu-DOTA-TATE, were enrolled. Safety was the primary objective, whereas effectiveness was a secondary objective. In this article, findings were analyzed and compared with the NETTER-1 and NETTER-2 trials. **Results:** Baseline characteristics were comparable to NETTER-1 and NETTER-2 except for the notably high proportion of G2 (77.1%) among participants. Less than half of patients (41.0%) completed four cycles of [^177^Lu]Lu-DOTA-TATE treatment, presenting a lower portion of completion rate compared to 75.7% in NETTER-1 and 87.8% in NETTER-2. Among the 83 patients, 60 patients (72.3%, 239 cases) had at least 1 AE, with 6 patients (7.2%, 8 cases) experiencing SAEs. The most common AE was nausea (34.9%, 46 cases), and most AEs were mild in severity (94.6%, 226 cases). Overall, the safety profile in this study presented minimal differences from NETTER-1 and NETTER-2. This study reports 37.7% of ORR which was between 14.7% in NETTER-1 and 43.0% in NETTER-2. **Conclusions:** This nationwide post-marketing surveillance study complemented the safety and effectiveness of [^177^Lu]Lu-DOTA-TATE among Koreans, which was not probed in two pivotal trials. The data would support the clinical implication of [^177^Lu]Lu-DOTA-TATE for the GEP-NETs treatment.

## Simple Summary

This open-label, non-interventional, primary data collection, multi-center, non-comparative, non-randomized observational nationwide post-marketing surveillance study was conducted in accordance with Ministry of Food and Drug Safety (MFDS) requirements to evaluate the safety and effectiveness of [^177^Lu]Lu-DOTA-TATE (Lutathera^TM^), a targeted radioligand therapy for gastroenteropancreatic neuroendocrine tumors (GEP-NETs). This post-marketing surveillance study presents the first real-world evidence on the safety and effectiveness results of [^177^Lu]Lu-DOTA-TATE in South Korea, and compares these findings with the safety and tolerability outcomes previously reported in two phase III clinical trials. Knowing a very small number of Asian patients enrolled into phase III trials, this first real-world evidence is expected to bridge a gap in safety data for Asian patients and eventually help physicians integrate [^177^Lu]Lu-DOTA-TATE into routine clinical practice. This also may enhance treatment strategies for patients with GEP-NETs in countries with healthcare systems similar to South Korea.

## 1. Introduction

Gastroenteropancreatic neuroendocrine tumors (GEP-NETs) are the most common subtype of NETs that originate within neuroendocrine cells [1,2,3], with an incidence rate of 3.56 per 100,000 according to the SEER 18 registry (2000–2012) [4,5,6]. Although GEP-NETs account for only 5.84% of all GEP tumors, their incidence is increasing at an annual rate of 4.5%, which is notably higher than the reported incidence of most other neoplasms [7,8,9,10]. According to the single-center study, the incidence of NETs among South Koreans was estimated to be 24.1 per 100,000, which is markedly higher than population-based estimates [11]. The hindgut is the most common site of origin of NETs (38.3%), followed by the pancreas (16.2%), foregut (13.5%), and midgut (4.9%) in the Korean population [12].

Lutathera^TM^ ([^177^Lu]Lu-DOTA-TATE) is the first radiopharmaceutical approved for peptide receptor radionuclide therapy (PRRT). PRRT is a promising and well-tolerated treatment modality that specifically binds to peptide receptor-positive tumors [13,14]. [^177^Lu]Lu-DOTA-TATE is a therapeutic radioligand that combines the radionuclide ^177^Lu (Lutetium) with the somatostatin analog DOTA-TATE, enabling targeted ionizing radiation to somatostatin receptor (SSTR)-positive tumor cells [15,16,17]. [^177^Lu]Lu-DOTA-TATE was approved by the European Medicines Agency (EMA) in 2017 and the U.S. Food and Drug Administration (FDA) in 2018 [17,18,19]. Ministry of Food and Drug Safety (MFDS), the health authority of South Korea, approved [^177^Lu]Lu-DOTA-TATE as an orphan drug for the treatment of SSTR-positive GEP-NETs in adults in July 2020. Although [^177^Lu]Lu-DOTA-TATE was approved in Korea based on a large-scale Phase III clinical trial, the absence of clinical data specifically evaluating its effectiveness in Korean patients remains a significant gap. While several other studies have included Asian populations, this study is the only one to prospectively observe adult patients in real-world practice in a non-interventional manner.

The NETTER-1 trial was an open-label, randomized, comparator-controlled, phase III trial conducted at 41 sites in 8 countries across Europe and the United States. Most patients were Caucasian/White, with 188 (82.1%), and Asian patients accounted for 1 (0.4%). A total of 231 patients with inoperable, somatostatin analog (SSA)-progressed, SSTR-positive, well-differentiated, G1 or G2 midgut NET were randomized into either [^177^Lu]Lu-DOTA-TATE 7.4 GBq (200 mCi) every 8 weeks up to four cycles with high-dose long-acting octreotide 30 mg ([^177^Lu]Lu-DOTA-TATE group) or high-dose long-acting octreotide 60 mg every 4 weeks (control group). The primary endpoint was met by showing that treatment with [^177^Lu]Lu-DOTA-TATE was associated with a statistically significant risk reduction of 82% (HR 0.18 [95% CI: 0.11, 0.29]; *p* < 0.0001) for disease progression or death compared to the control group. Also, [^177^Lu]Lu-DOTA-TATE was associated with limited acute toxic effects [20].

The NETTER-2 trial was an open-label, randomized, parallel-group, superiority, phase III trial conducted at 38 centers in 9 countries across North America, Europe, and Asia. Most patients were White with 165 (73.0%), while Asian patients accounted for 34 (15.0%), including 31 (13.7%) Koreans and 3 (1.3%) Indians. A total of 226 patients with newly diagnosed, advanced, SSTR-positive, well-differentiated, G2 or G3 GEP-NETs were randomized either to either [^177^Lu]Lu-DOTA-TATE group or the control group (same group characteristics as in the NETTER-1 trial). This study demonstrated significant improvements in progression-free survival and response rates with [^177^Lu]Lu-DOTA-TATE with high-dose long-acting octreotide compared to somatostatin analogs alone. No study drug-related deaths were reported during the treatment period [21].

NETTER-1 and NETTER-2 trials, extensively detailed in previous publications [20,21,22], provide robust evidence of [^177^Lu]Lu-DOTA-TATE ‘s clinical benefits and safety. Since both trials primarily enrolled patients from Europe and the United States, and only a small number of Asians joined the NETTER-2, evaluating the real-world effect of [^177^Lu]Lu-DOTA-TATE in the Asian population is limited.

As a part of the Risk Management Plan (RMP) regulated by MFDS, this post-marketing surveillance study aimed to investigate adverse events that may not have been observed during the development of [^177^Lu]Lu-DOTA-TATE, as well as factors influencing its safety and effectiveness in real-world practice. Conducted across six institutions in South Korea, this study monitored all patients treated with [^177^Lu]Lu-DOTA-TATE for safety and effectiveness, specifically in terms of adverse event occurrence and expected effectiveness to fulfill MFDS requirements.

This is the first real-world evidence on the safety and effectiveness report of [^177^Lu]Lu-DOTA-TATE in South Korea, with a substantial number of patients comparable to those of the NETTER-1 and NETTER-2 trials. The findings are compared to identify potential differences in treatment outcomes within the Korean population, providing valuable insights for optimizing care for GEP-NETs.

## 2. Materials and Methods

### 2.1. Study Design

This multicenter observational study was conducted to evaluate the safety and effectiveness of [^177^Lu]Lu-DOTA-TATE (Advanced Accelerator Applications s.r.l/Ivrea/Italy, Advanced Accelerator Applications Iberica, S.L.U/Zaragoza/Spain) in GEP-NET patients in South Korea. Patients were treated according to the locally approved label and routine medical practice. Data were collected through case report forms as per the protocol. For each patient, the last observation was approximately 30 days from the final [^177^Lu]Lu-DOTA-TATE treatment.

### 2.2. Study Population

From July 2020 to July 2024, a total of 89 patients were enrolled across six centers in South Korea. Of these, 83 patients who received at least one dose of [^177^Lu]Lu-DOTA-TATE were included in the safety analysis set, excluding 6 patients who did not receive the drug. The effectiveness was analyzed among 61 patients, excluding 22 patients whose medical records were insufficient for the evaluation. Patients diagnosed with GEP-NETs and prescribed [^177^Lu]Lu-DOTA-TATE in accordance with the approved local label, administering 7.4 GBq up to four times at 8-week intervals, in South Korea, were eligible for this study.

For reference, the NETTER-1 trial included a full analysis set (FAS) composed of 229 patients, 116 in the [^177^Lu]Lu-DOTA-TATE group and 113 in the control group, while the safety set (SAF) composed of 223 patients, 112 in the [^177^Lu]Lu-DOTA-TATE group and 111 in the control group.

The NETTER-2 trial included a FAS composed of 226 patients, 151 in the [^177^Lu]Lu-DOTA-TATE group and 75 in the control group, while the SAF included 220 patients, 147 in the [^177^Lu]Lu-DOTA-TATE group and 73 in the control group. The results of the NETTER-1 and NETTER-2 trials were based on their respective clinical study reports.

### 2.3. Assessments

The primary endpoint was safety, measured by the incidence of adverse events (AEs). The secondary endpoint was effectiveness, represented as objective response rate (ORR), assessed by the investigator.

Safety assessment included the incidence rate, number of events, and details regarding all AE/adverse drug reaction (ADR), serious adverse event (SAE)/serious adverse drug reaction (SADR), and unexpected AE/ADR. Patients were monitored for AEs up to 30 days from the last dose of [^177^Lu]Lu-DOTA-TATE. The study also collected the type of AE, start and end dates, severity (mild, moderate, severe), causality as assessed by the investigator, actions taken, outcomes, and expectedness as assessed by the investigator and/or sponsor. AEs were coded by preferred terms using the Medical Dictionary for Regulatory Activities (MedDRA) version 27.0.

ORR was the surrogate for an effectiveness assessment. The ORR was defined as the proportion of patients achieving either a partial response (PR) or complete response (CR) based on the Response Evaluation Criteria in Solid Tumors (RECIST) version 1.1 [23]. The tumor was assessed via CT or MRI by the investigator as a part of routine clinical practice. As a non-interventional study, no additional diagnostic or monitoring procedures outside of standard care were applied. All assessments following [^177^Lu]Lu-DOTA-TATE injection were performed at the discretion of the investigator based on clinical necessity.

In the NETTER-1 trial, objective CT/MRI tumor assessments were conducted every 12 ± 1 weeks for 72 weeks from the randomization date and centrally confirmed by the Independent Review Committee (IRC). Patients who completed 76 weeks or more of treatment/assessment discontinued treatment but continued 6-monthly assessments for a total period of 5 years from the date of randomization of the last patient.

In the NETTER-2 trial, CT/MRI tumor assessments were performed at Week 16, Week 24, and then every 12 ± 1 weeks thereafter. This study incorporated central, blinded, real-time IRC CT/MRI assessment, with tumor response images assessed both centrally and by the site’s local radiologist. Additional tumor assessment data were collected for up to 3 years after the end of the Treatment Phase of the last subject. During the follow-up phase, RECIST 1.1 assessments were performed locally every 6 months ± 1 month.

### 2.4. Statistical Analysis

Statistical analysis of all data was performed using SAS^®^ statistical software 9.4 (SAS Institute, Cary, NC, USA). Descriptive analyses were used to summarize quantitative data and sample characteristics. Continuous variables were reported as the number of observations, mean, standard deviation (SD), minimum, maximum, and 95% confidence interval (CI). Categorical variables were summarized as the number and proportion of patients with observed data.

## 3. Results

### 3.1. Baseline Characteristics

The average age of patients was 59.3 ± 11.6 years, with a median age of 60 years (range: 22 to 85 years). Half of the patients were female (50.6%) and the other half were male (49.4%). The grade of SSTR expression was evaluated by the PET-based Krenning Score. Most patients presented with grade 4 SSTR expression (67.5%), followed by grade 3 (31.3%). No patients had grade 1 or 2 expression. The most frequent NET grade was G2 (77.1%), followed by G1 (15.7%) and G3 (7.2%); Table 1.

When compared to NETTER-1 and NETTER-2 trials, the baseline characteristics in this study were comparable, with a notable predominance of G2 NET grade (77.1%) in Table 2.

### 3.2. Treatment Parameters of [^177^Lu]Lu-DOTA-TATE

In this study, a total of 34 patients (41.0%) received all four cycles of [^177^Lu]Lu-DOTA-TATE, followed by 20 patients (24.1%) receiving two cycles, 16 patients (19.3%) receiving one cycle, and 13 patients (15.7%) receiving three cycles. The mean treatment duration was 118.2 ± 77.2 days, with a mean administered dose (GBq) of 20.6 ± 8.8 GBq.

Dose changes or interruptions were reported in 20 patients (24.1%, including 2 patients who were double-counted), including permanent interruptions in 17 patients (20.5%), temporary interruptions in 2 patients (2.4%), and dose adjustments in 1 patient (1.2%). The primary reasons for dose changes or interruptions were categorized as “other” (9 patients, 10.8%), encompassing 7 cases of end of the study period, 1 case of follow-up loss, and 1 case of consent withdrawal in Table 3.

When compared to NETTER-1 and NETTER-2, dose exposure was comparable. However, a lower percentage of patients completed all four cycles of treatment (41.0%; 34/83 patients) in this study than in NETTER-1 and NETTER-2 (75.7%; 84/112 patients and 87.8%; 129/147 patients, respectively, Table 4).

### 3.3. Safety

A total of 60 patients (72.3%, 239 cases) had at least one AE, predominantly mild (94.6%, 226 cases), followed by moderate (3.4%, 8 cases) and severe (2.1%, 5 cases). ADRs were observed in 49 patients (59.0%, 150 cases), with ADRs in 47 patients (56.6%, 146 cases) being [^177^Lu]Lu-DOTA-TATE-related.

The most common AEs were nausea (34.9%, 46 cases), alopecia (14.5%, 15 cases), fatigue (13.3%, 15 cases), abdominal pain (9.6%, 14 cases), and dizziness (9.6%, 8 cases). The most frequently reported ADRs related to [^177^Lu]Lu-DOTA-TATE were nausea (32.5%, 44 cases), alopecia (14.5%, 15 cases), fatigue (9.6%, 10 cases), and dyspepsia (7.2%, 8 cases). Regarding hematologic toxicity, thrombocytopenia was reported in 3.6% (4 cases), and pancytopenia was reported in 1.2% (1 case), both of which were ADRs related to [^177^Lu]Lu-DOTA-TATE. All cases of thrombocytopenia were non-serious (non-SAE), whereas pancytopenia was reported as an SAE.

SAEs were reported in six patients (7.2%, 8 cases), of which two patients (2.4%, 4 cases) experienced SADRs related to [^177^Lu]Lu-DOTA-TATE, including abdominal pain, diarrhea, vomiting, and pancytopenia in 1.2% (1 case) each, respectively, in Table 5.

Safety findings were consistent with those reported in NETTER-1 and NETTER-2, although this study exhibited a higher proportion of mild AEs and fewer severe events.

In NETTER-1, AEs were reported in 110 patients (98.2%), with 57.2% categorized as grade 3 or higher in severity. In NETTER-2, AEs were reported in 101 patients (68.7%), with 15.6% being grade 3 or higher. The most common AEs in NETTER-1 were nausea (64.3%), vomiting (52.7%), fatigue (37.5%), abdominal pain (25.9%), and diarrhea (25.9%), while in NETTER-2, the most common AEs were nausea (20.4%), fatigue (14.3%), alopecia, asthenia, and anemia (13.6% each, respectively).

In NETTER-1 and NETTER-2, 102 patients (91.1%) and 96 patients (65.3%) showed treatment-emergent ADR related to the study medication, respectively, in Table 6.

### 3.4. Effectiveness

A secondary endpoint, ORR, was clinically assessed by the investigator. Among 61 patients in the effectiveness analysis set, the ORR was 37.7%, with 34 patients (55.7%) in stable disease (SD), 23 patients (37.7%) in PR, and 4 patients (6.6%) in progressive disease (PD). No cases of CR were observed. When assessed by radiologic imaging (CT or MRI), the ORR was 27.3% in Table 7.

The ORR results in this study were comparable to NETTER-1 and NETTER-2, though difference was observed due to assessment methods and patient characteristics. In the [^177^Lu]Lu-DOTA-TATE group, the ORR was 14.7% and 43.0% in NETTER-1 and NETTER-2, respectively. Both studies demonstrated a significant improvement in ORR for patients treated with [^177^Lu]Lu-DOTA-TATE compared to the control group (4.0% and 9.3%, respectively, Table 8).

## 4. Discussion

This post-marketing surveillance study provides insights into the safety and effectiveness of [^177^Lu]Lu-DOTA-TATE in South Korea, marking the first large-scale, real-world evidence of [^177^Lu]Lu-DOTA-TATE involving Korean patients with GEP-NETs. Moreover, we compared these findings with the safety, tolerability, and effectiveness outcomes reported in two pivotal clinical trials (NETTER-1 and NETTER-2), providing clinically relevant implications for physicians utilizing this treatment in routine clinical practice.

The patient characteristics in this study were generally comparable to those in NETTER-1 and NETTER-2, with the exception of the NET grade distribution. In this study, the majority of patients had a NET grade of G2 (77.1%), with some patients having G1 and G3. In contrast, the NETTER-1 study included the highest proportion of patients with G1 (65.5%) and no patients with G3, whereas the NETTER-2 study had the highest proportion of patients with G2 (65.6%) and no patients with G1. Notably, only 40% of patients completed the planned four cycles of treatment. While the average total administered dose (in GBq) was within the expected range, this completion rate (41%) is remarkably lower than the 75.7% observed in NETTER-1 and the 87.8% in NETTER-2. [^177^Lu]Lu-DOTA-TATE is covered by national health insurance in South Korea as a third-line treatment for gastrointestinal NETs and fourth-line for pancreatic NETs. The authors attributed this discrepancy in the percentage of treatment completion to the advanced disease stages (stage 3 or 4) of most patients receiving [^177^Lu]Lu-DOTA-TATE under Korean insurance conditions, as well as the associated poor health status, since patients who received other interventions before the treatment had been enrolled. Disease progression and clinical deterioration during treatment are believed to have hindered the completion of all four cycles.

The most frequently reported AEs were nausea, alopecia, fatigue, abdominal pain, and dizziness, consistent with findings from NETTER-1 and NETTER-2. GEP-NETs often present with non-specific symptoms such as pain, nausea, and vomiting, and, in some cases, anemia due to intestinal blood loss [24,25]. The majority of AEs in this study were mild, and no new safety signals were identified. Given the known hematologic toxicity of this drug [26], thrombocytopenia occurred in 3.6% of patients and pancytopenia in 1.2%, both at lower rates than those reported in the two pivotal studies (thrombocytopenia: 13.4% in NETTER-1 and 6.8% in NETTER-2; pancytopenia: 3.6% in NETTER-1). Despite the lower incidence, the risk of hematologic toxicities, including thrombocytopenia, lymphopenia, anemia, and pancytopenia, remains a concern due to the myelotoxic effects of the drug [27,28,29]. Therefore, careful monitoring is recommended throughout the treatment period.

The ORR in this study was 37.7%, which is higher than 14.7% reported in NETTER-1 but lower than 43.0% observed in NETTER-2. Differences in the patient populations between the two pivotal studies likely contributed to these variations. NETTER-1 involved patients with advanced, SSA-progressed, grade 1–2 midgut NETs, whereas NETTER-2 involved newly diagnosed patients with grade 2–3 advanced GEP-NETs and a Ki-67 index of 10–55%. Despite these differences, the ORR observed in this real-world study remains significant, particularly when compared to a previous Korean study on advanced NETs, which reported an ORR of 20.3% [30]. These findings underscore the clinical effectiveness of [^177^Lu]Lu-DOTA-TATE in treating SSTR-positive GEP-NETs among Korean patients.

Our study contributes to the growing body of evidence supporting the use of [^177^Lu]Lu-DOTA-TATE in real-world settings, particularly for Korean patients with SSTR-positive GEP-NETs. However, several limitations should be noted. First, as a non-interventional, real-world post-marketing surveillance study, the enrolled patients exhibited heterogeneity, and the overall follow-up period was limited to 30 days after the completion of administration. This limited observation period precluded the long-term monitoring of safety and effectiveness outcomes and may have hindered the detection of delayed adverse events. Patients in this study received both [^177^Lu]Lu-DOTA-TATE and Lysakare^TM^, a kidney-protective agent against radiation damage. While this paper did not specifically address Lysakare^TM^-related adverse events, it cannot be excluded that adverse events may have occurred due to Lysakare^TM^. Additionally, the sample size (*n* = 83) was smaller than that of the two pivotal studies (NETTER-1, *n* = 116; NETTER-2, *n* = 151), and direct comparisons with interventional studies are inherently limited. Further investigations are required to address these limitations in future studies.

## 5. Conclusions

This post-marketing surveillance study provides the first real-world evidence on the safety and effectiveness of [^177^Lu]Lu-DOTA-TATE in South Korean patients with GEP-NETs. The safety profile observed in this study was consistent with those reported in the NETTER-1 and NETTER-2 trials, with no new safety concerns identified. The ORR 37.7% which falls between 14.7% in NETTER-1 and 43.0% NETTER-2, is meaningful as the study population reflects the eligibility criteria of both trials.

Overall, this study highlights the therapeutic value of [^177^Lu]Lu-DOTA-TATE in the South Korean population, demonstrating both an acceptable safety profile and clinically meaningful effectiveness. Despite not being evaluated in two pivotal trials, this study underscores the clinical utility of [^177^Lu]Lu-DOTA-TATE in routine practice for patients with SSTR-positive GEP-NETs in South Korea.

## Figures and Tables

**Table 1:** Baseline demographics and characteristics.

Characteristic	Post-Marketing Surveillance (SAP, *n* = 83)
**Age (year, mean ± SD)**	59.3 ± 11.6
Median (min–max)	60.0 (22.0–85.0)
>65 age, *n* (%)	31 (37.4)
**Sex, *n* (%)**	
Male	41 (49.4)
Female	42 (50.6)
**GEP-NETs duration (month, mean ± SD)**	67.8 ± 59.9
Median (min–max)	55 (1.6–309.0)
**SSTR grade, *n* (%)**	
Grade 1	0 (0.0)
Grade 2	0 (0.0)
Grade 3	26 (31.3)
Grade 4	56 (67.5)
**Ki67 index (mean ± SD)**	10.3 ± 11.2
Median (min–max)	6 (0.1–61.7)
**Mitotic count (mean ± SD)**	3.7 ± 4.3
Median (min–max)	3 (0.0–20.0)
**NET grade, *n* (%)**	
Grade 1	13 (15.7)
Grade 2	64 (77.1)
Grade 3	6 (7.2)

GEP-NET, gastroenteropancreatic neuroendocrine tumor; SSTR, somatostatin receptor; NET, neuroendocrine tumor; SAP, safety analysis population. Plus-minus values are means ± standard deviation. Missing (number of patients): SSTR Grade (1), Ki-67 index (6), mitotic count (42).

**Table 2:** Summary of baseline characteristics in NETTER-1 and NETTER-2.

Characteristic	NETTER-1 (FAS, *n* = 116)	NETTER-2 (FAS, *n* = 151)
**Age (year, mean ± SD)**	63.4 ± 9.4	60.2 ± 13.2
Median	64.0	61.0
**Sex, *n* (%)**		
Male	63 (54.3)	81 (53.6)
Female	53 (45.7)	70 (46.4)
**SSTR grade ^(1)^, *n* (%)**		
Grade 1	0 (0.0)	0 (0.0)
Grade 2	11 (9.5)	0 (0.0)
Grade 3	35 (30.2)	56 (37.1)
Grade 4	70 (60.3)	95 (62.9)
**NET grade, *n* (%)**		
Grade 1	76 (65.5)	-
Grade 2	40 (34.5)	99 (65.6)
Grade 3	-	52 (34.4)
**Ki67 index (mean ± SD)**		19.7 ± 10.1
Median		17.0
ENETS G1: ≤2% positive tumor cells	76 (65.5)	
ENETS G2: 3–20% positive tumor cells	40 (34.5)	

SSTR, somatostatin receptor; NET, neuroendocrine tumor; ENETS, European Neuroendocrine Tumour Society; FAS, full analysis set. Plus-minus values are means ± standard deviation. (1) Centralized Octreoscan Tumour Uptake score (highest score) in NETTER-1. Highest SSTR tumor update score is based on the cancer diagnosis in NETTER-2.

**Table 3:** Treatment exposure with [^177^Lu]Lu-DOTA-TATE.

	Post-Marketing Surveillance (SAP, *n* = 83)
**Number of administrations, *n* (%)**	
1 cycle	16 (19.3)
2 cycles	20 (24.1)
3 cycles	13 (15.7)
4 cycles	34 (41.0)
Total	83
**Duration of exposure (days, mean ± SD)**	118.2 ± 77.2
**Mean total dose (GBq, mean ± SD)**	20.6 ± 8.8
**Mean dose per administration (GBq, mean ± SD) ^(1)^**	7.4 ± 0.2
**Cumulative Dose (GBq, mean ± SD)**	44.0 ± 27.4
**Dose changes or interruptions ^(2)^, *n* (%)**	
Dose changes	1 (1.2)
Temporary interruptions	2 (2.4)
Permanent interruptions	17 (20.5)
Not applicable	76 (91.6)
**Reason for dose changes/interruption ^(2)^, *n* (%)**	
Physician decision	3 (3.6)
Withdrew consent	0 (0.0)
Non-compliance	2 (2.4)
Adverse events	5 (6.0)
Other	9 (10.8)

SAP, safety analysis population. Plus–minus values are means ± standard deviation. (1) Mean dose per administration (GBq) = total dose/total number of administrations. (2) Double-counted

**Table 4:** Summary of dose exposure with [^177^Lu]Lu-DOTA-TATE in NETTER-1 and NETTER-2.

	NETTER-1 (SAF, *n* = 112)	NETTER-2 (SAF, *n* = 147)
**Number of administrations, *n* (%)**		
1 cycle	6 (5.4)	1 (0.7)
2 cycles	12 (10.8)	10 (6.8)
3 cycles	9 (8.1)	7 (4.8)
4 cycles	84 (75.7)	129 (87.8)
Total	111 **^(2)^**	147
**Mean total dose (GBq)**	25.6	-
**Mean dose per administration (GBq, mean ± SD) ^(1)^**	6.7	7.3 ± 0.4
**Cumulative Dose (GBq, mean ± SD)**	-	27.6 ± 4.6
**Duration of exposure (weeks, mean ± SD)**	-	31.1 ± 5.0

SAF, safety set. Plus–minus values are means ± standard deviation. The SAF consisted of all randomized patients who received at least one dose of study drug in NETTER-1 and all patients who received at least one dose of study treatment in NETTER-2. (1) Mean dose per administration means single dose total dose (GBq) in NETTER-1 and dose per administration (GBq/cycle) in NETTER-2. (2) The total number of 111 patients received [^177^Lu]Lu-DOTA-TATE treatment in SAF.

**Table 5:** AE, ADR, and SAE findings.

Event	Post-Marketing Surveillance (SAP, *n* = 83)	
	Total	[^177^Lu]Lu-DOTA-TATE-Related
**Adverse event (AE) (%), (case)**	60 (72.3), (239)	
Severity **^(1)^**		
Mild	94.6 (226)	
Moderate	3.4 (8)	
Severe	2.1 (5)	
Total case	100.0 (239)	
**Adverse drug reaction (ADR) (%), (case)**	49 (59.0), (150)	47 (56.6), (146) **^(3)^**
Severity **^(1)^**		
Mild	95.3 (143)	
Moderate	3.3 (5)	
Severe	1.3 (2)	
Total case	100.0 (150)	
**Serious adverse event (SAE) (%), (case)**	6 (7.2), (8)	
**Serious adverse drug reaction (SADR) (%), [case]**	2 (2.4), (4)	2 (2.4), (4)
**Unexpected AE (%), (case)**		19 (22.9), (35)
**Unexpected ADR (%), (case)**		5 (6.0), (6)
**Unexpected SAE (%), (case)**		1 (1.2), (1)
**Unexpected SADR (%), (case)**		0 (0.0), (0)
**The most frequent (≥5%) AE (%), (case)**		
Nausea	29(34.9), (46)	27(32.5), (44)
Alopecia	12(14.5), (15)	12(14.5), (15)
Fatigue	11(13.3), (15)	8(9.6), (10)
Abdominal Pain	8(9.6), (14)	5(6.0), (9)
Dizziness	8 (9.6), (8)	5 (6.0), (5)
Dyspepsia	7 (8.4), (10)	6 (7.2), (8)
Vomiting	7 (8.4), (8)	5 (6.0), (6)
Decreased appetite	5 (6.0), (5)	4 (4.8), (4)
Diarrhea	6 (7.2), (6)	4 (4.8), (4)
Platelet count decreased	5 (6.0), (5)	5 (6.0), (5)
**Types of SAE reported in the PMS (%), (case)**		
Abdominal pain	2 (2.4), (2)	1 (1.2), (1)
Diarrhea	1 (1.2), (1)	1 (1.2), (1)
Vomiting	1 (1.2), (1)	1 (1.2), (1)
Disseminated intravascular coagulation	1 (1.2), (1)	0 (0.0), (0)
Pancytopenia	1 (1.2), (1)	1 (1.2), (1)
Neuroendocrine tumour **^(2)^**	1 (1.2), (1)	0 (0.0), (0)
Acute kidney injury	1 (1.2), (1)	0 (0.0), (0)

SAP, safety analysis population. Data are n (%), (case) All notations conform to preferred term of MedDRA version 27.0. (1) Severity criteria were referred to “Guideline on re-examination affairs of new drug, etc.” published by the MFDS. Data are number of AEs or ADRs, percentages, and individual events. (2) The original reported verbatim for this adverse event is “progression of neuroendocrine tumor”, and it is presented as the preferred term “neuroendocrine tumour” in PMS report. (3) [^177^Lu]Lu-DOTA-TATE-related ADRs were defined as having a causal relationship with [^177^Lu]Lu-DOTA-TATE as certain, probable/likely, possible, conditional/unclassified, or unassessable/unclassifiable.

**Table 6:** Summary of safety findings in NETTER-1 and NETTER-2.

	NETTER-1 (SAF, *n* = 112)	NETTER-2 (SAF, *n* = 147)
**Adverse event (AE) ^(1)^** (%)	110 (98.2)	101 (68.7)
Severity **^(2)^**		
Grade 1 (Mild)	11 (9.8)	78 (53.1) *
Grade 2 (Moderate)	35 (31.3)	
Grade 3 (Severe)	51 (45.5)	23 (15.6) *
Grade 4 (Threatening/Disabling)	6 (5.4)	
Grade 5 (Death)	7 (6.3)	
**Adverse drug reaction (ADR)** (%)	102 (91.1)	96 (65.3)
Severity **^(2)^**		
Grade 1 (Mild)	24 (21.4)	74 (50.3) *
Grade 2 (Moderate)	44 (39.3)	
Grade 3 (Severe)	30 (26.8)	22 (15.0) *
Grade 4 (Threatening/Disabling)	4 (3.6)	
Grade 5 (Death)	0 (0.0)	
**Serious adverse event (SAE)**	35 (31.3)	8 (5.4)
**Serious adverse drug reaction (SADR)**	13 (11.6)	8 (5.4)
**The most frequent (≥10%) AE reported in the [^177^Lu]Lu-DOTA-TATE group**		
Nausea	72 (64.3)	30 (20.4)
Vomiting	59 (52.7)	<10%
Fatigue	42 (37.5)	21 (14.3)
Abdominal pain	29 (25.9)	<10%
Diarrhea	29 (25.9)	18 (12.2)
Decreased appetite	23 (20.5)	<5%
Dizziness	19 (17.0)	<5%
Headache	19 (17.0)	<5%
edema peripheral	18 (16.1)	<5%
Abdominal distension	18 (16.1)	<5%
Alopecia	16 (14.3)	20 (13.6)
Back pain	14 (12.5)	-
Platelet count decreased	13 (11.6)	19 (12.9)
Lymphocyte count decreased	12 (10.7)	15 (10.2)
Arthralgia	12 (10.7)	<5%
Pain in extremity	12 (10.7)	-
Asthenia	<10%	20 (13.6)
Anemia	-	20 (13.6)
White blood cell count decreased	<10%	18 (12.2)

SAF, safety set. * NETTER-2 classified adverse events into all grades and grade 3 or higher. Adverse events of grade 2 or less are defined as grade 2, and adverse events of grade 3 or higher are defined as grade 3. Data are n (%), (case). No number of events was suggested in NETTER-2. (1) Number of patients, and percentage, with at least one treatment emergent adverse event in NETTER-1, and number of patients, and percentage, related to any treatment AEs in NETTER-2. (2) Severity was defined as the maximum severity according to CTCAE classification version 4.03 in the NETTER-1 and as the number of patients with grade ≥ 3 AEs in NETTER-2.

**Table 7:** Objective response rate of patients treated with [^177^Lu]Lu-DOTA-TATE.

	Post-Marketing Surveillance (EAP, *n* = 61)
**Objective response rate (ORR) ^(1)^, *n* (%), (95% CI)**	23 (37.7), (25.6,51.0)
Complete response (CR)	0 (0.0)
Partial response (PR)	23 (37.7)
Stable disease (SD)	34 (55.7)
Progressive disease (PD)	4 (6.6)
**Objective response rate (ORR) ^(2)^, *n* (%), (95% CI)**	3(27.3), (6.0,61.0)
Complete response (CR)	0 (0.0)
Partial response (PR)	3 (27.3)
Stable disease (SD)	7 (63.6)
Progressive disease (PD)	1 (9.1)

EAP, effectiveness analysis population. (1) ORR results assessed clinically by investigators based on RECIST 1.1 criteria. (2) ORR results based on tumor assessment using CT or MRI. Missing (50).

**Table 8:** Objective response rate in [^177^Lu]Lu-DOTA-TATE group in NETTER-1 and NETTER-2.

	NETTER-1 (FAS, *n* = 116)	NETTER-2 (FAS, *n* = 151)
**Objective response rate (ORR) ^(1)^,** ***n* (%), (95% CI)**	15 (14.7), (7.8, 21.6)	65 (43.0) (35.0, 51.3)
**Best overall response, *n* (%)**		
Complete response (CR)	1 (0.9)	8 (5.3)
Partial response (PR)	14 (12.1)	57 (37.7)
Stable disease (SD)	80 (69.0)	72 (47.7)
Progressive disease (PD)	7 (6.0)	8 (5.3)
Not available **^(2)^**	14 (12.1)	6 (4.0)

FAS, full analysis set. (1) The ORR analysis results were based on central assessment by the Independent Review Committee in NETTER-1 and on central review using RECIST 1.1 criteria in NETTER-2. (2) Missing or unknown.

## Data Availability

Data are contained within the article.

## Abbreviations

The following abbreviations are used in this manuscript:

GEP-NETs	Gastroenteropancreatic Neuroendocrine Tumors
PRRT	Peptide receptor radionuclide therapy
SSTR	Somatostatin receptor
EMA	European Medicines Agency
FDA	Food and Drug Administration
MFDS	Ministry of Food and Drug Safety
SSA	Somatostatin analog
RMP	Risk management plan
SAF	Safety set
AEs	Adverse events
ORR	Objective response rate
ADR	Adverse drug reaction
SAE	Serious adverse event
SADR	Serious adverse drug reaction
MedDRA	Medical Dictionary for Regulatory Activities
PR	Partial response
CR	Complete response
RECIST	Response Evaluation Criteria in Solid Tumors
IRC	Independent review committee
SD	Standard deviation
CI	Confidence interval
SAP	Safety analysis population
ENETS	European Neuroendocrine Tumour Society
FAS	Full analysis set
SD	Stable disease
PD	Progressive disease
